# Clinical study to evaluate the safety and effectiveness of the Aesculap Activ-L™ artificial disc in the treatment of degenerative disc disease

**DOI:** 10.1186/1471-2482-10-14

**Published:** 2010-04-09

**Authors:** James J Yue, Fred F Mo

**Affiliations:** 1 Department of Orthopaedics and Rehabilitation, Yale University School of Medicine, 800 Howard Ave, New Haven, CT06511, USA

## Abstract

**Background:**

The objective of this clinical study is to evaluate the safety and effectiveness of the Activ-L Artificial Disc for treatment of single-level degenerative disc disease of the lumbar spine in patients who have been unresponsive to at least six months of prior conservative care. The hypothesis of the study is that the Activ-L Disc is non-inferior to the control (the Charité^® ^Artificial Disc [DePuy Spine] or ProDisc-L^® ^Total Disc Replacement [Synthes Spine]) with respect to the rate of individual subject success at 24 months. Individual subject success is a composite of effectiveness and safety.

**Methods/Design:**

The study proposed is a prospective, randomized, single-masked, controlled, multi-center clinical trial consisting of an estimated 414 subjects with single-level DDD of the lumbar spine (L4/L5, or L5/S1) who have failed to improve with conservative treatment for at least six months prior to enrollment. After enrollment, subjects will be randomized in a 2:1 ratio to either the Activ-L Disc (investigational device) or the control (Charité or ProDisc-L). Radiographic endpoints will be evaluated by an independent reviewer at an imaging core laboratory. Each subject will be followed for 5 years post-treatment.

**Discussion:**

The safety and effectiveness of the Activ-L Artificial Disc for treatment of single-level degenerative disc disease of the lumbar spine will be equivalent to Charité^® ^Artificial Disc [DePuy Spine] or ProDisc-L^® ^Total Disc Replacement [Synthes Spine] at 24 months.

**Trial Registration:**

Current Controlled Trials NCT00589797.

## Background

Lower back pain is a leading cause of physician patients report back pain annually in the U.S. Conservative treatment ultimately fails in over 4.5 million patients, and of these, some 500,000 undergo surgery of the lumbar spine, of which 200,000 are fusions [1-5].

One of the primary causes of lower back pain is degeneration of the intervertebral disc. Disc degeneration may result in rupture or herniation, spinal instability, articular facet syndrome, or painful impingement on the nerves enclosed in the spinal visits in the United States, second only to the common cold. The resultant pain may also lead to significant disability.

Spinal fusion effectively eliminates the motion segment between two vertebrae by use of a bone graft, thereby providing improved stability and decreased pain. The success rate in spinal fusion has proven highly variable; averaging approximately 60 to 70% [2]. The use of internal fixation generally increases the fusion rate but also increases the stiffness of the fused area. This may lead to increased stress on the adjacent nonfused segments and, in the case of L5/S1 fusions, on the sacroiliac joint.

Moreover, in addition to the potential failure to achieve fusion, the possibility of long-term adverse effects of fusion has been well documented in the literature. Long-term complications include bone graft donor site pain, pseudoarthrosis, spinal stenosis, spondylolysis acquisita, and, as a result of the increased stress on the adjacent spinal segments and the sacroiliac joint, accelerated degenerative changes in the disc and facet joints [1]. Segments adjacent to the spinal fusion may also manifest disc herniation, degeneration, spinal stenosis, spondylolysis, facet joint arthritis, or instability. The incidence of the adverse effects reported in the literature is significant, although highly variable. As many as one third of the patients who undergo fusion may develop significant short-term or long-term post-surgical complications [3].

The Activ-L, ProDisc-L and Charité devices are designed to provide a treatment modality as an alternative to fusion. These devices are modular systems and are intended to be used as intervertebral dynamic disc spacers in the lumbar vertebral region at the L3/L4 (ProDisc-L only), L4/L5 and L5/S1 levels. Disc replacement devices have the advantage of preserving spinal motion thus potentially avoiding adjacent level disease and complications secondary to lack of solid fusion.

The ProDisc-L and Charité devices consist of two endplates composed of a CoCrMo alloy and an ultra high molecular weight polyethylene (UHMWPE) core. The endplates are available in a range of endplate and core sizes and endplate lordosis angles. All ProDisc endplates are plasma sprayed with calcium phosphate and titanium alloy. The Charité device has both coated and uncoated endplates. The devices are modular so the surgeon can select the size and angulation that best fits the patient's unique anatomic and physiologic requirements. The Charité CoCrMo endplates each have six fixation "teeth" on each side of the endplate for attachment to the adjacent vertebral bodies while the ProDisc-L has a central keel and two lateral spikes [6].

The Charité UHMWPE core is a convex bearing surface that fits into a circular groove in the endplate. The mobile design provides a floating center of rotation. The ProDisc design incorporates a semi-constrained ball and socket joint. These devices are designed so that the endplates are inserted and secured without the core. After endplate placement, the disc space is distracted to allow the core to be seated in the inferior endplate. By comparison, the Activ-L is inserted as a single unit and therefore does not require this second distraction step. This difference in insertion method is intended to reduce the risk of over-distraction.

## Methods/Design

The following study was reviewed and approved by the Yale University Human Investigation Committee.

**Figure 1:**
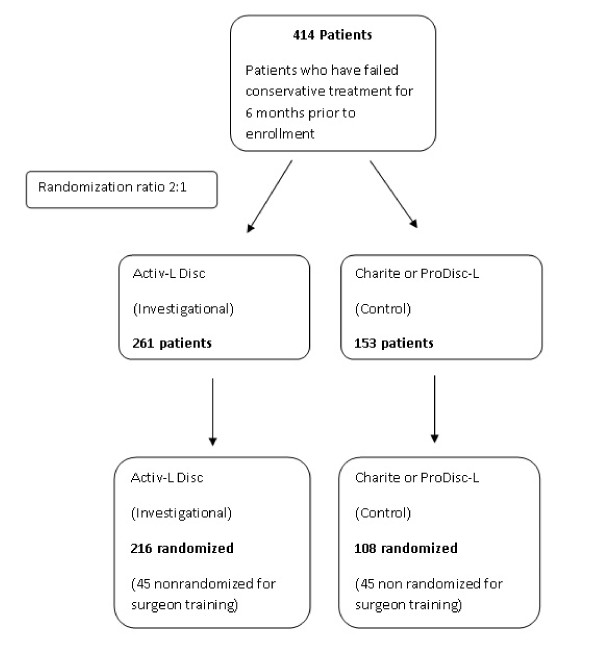
**Patient progression through both arms of trial**.

The study proposed is a prospective, randomized, single-masked, controlled, multi-center clinical trial consisting of an estimated 414 subjects with single-level DDD of the lumbar spine (L4/L5, or L5/S1) who have failed to improve with conservative treatment for at least six months prior to enrollment. After enrollment, subjects will be randomized in a 2:1 ratio to either the Activ-L Disc (investigational device) or the control (Charité or ProDisc-L). Each investigational site will have a separate, blocked randomization schedule. Fifteen (15) to 20 investigational sites will participate in the investigation. Sites will be encouraged to randomize a minimum of 10 subjects per site and to implant a minimum of four control devices and sites are bound by a maximum of 64 subjects. The first three subjects at each investigational site will receive the Activ-L Disc and will be not be randomized, these patients will be outside of the trial. The purpose of these procedures will be for Surgeon training. Investigators who have already undergone training in implantation of the control disc will be selected for participation; however, for any participating investigator who has not performed at least three prior control implantations, up to three nonrandomized control implantations may also be performed prior to enrolling randomized subjects into the study. This will ensure that experience and proficiency with the implantation method for both discs is comparable. Thus, the maximum total enrollment in the study, including both randomized and nonrandomized cases, is 414 consisting of 261 Activ-L (216 randomized and 45 nonrandomized) and 153 controls (108 randomized and 45 nonrandomized controls). Please refer to figure 1.

The hypothesis of the study is that the Activ-L Disc is non-inferior to the control (ProDisc-L or Charité) with respect to the rate of individual subject success at 24 months. Individual subject success is a composite of effectiveness and safety.

The null hypothesis for the investigation is that the success rate in the Activ-L group is more than 15% lower than the success rate in the control group. The alternative hypothesis is that the success rate in the Activ-L group is no more than 15% lower than the success rate in the control group. The hypotheses will be evaluated according to the method of Blackwelder [7].

### Inclusion Criteria

• Age 18 - 60 years and skeletally mature

• Symptomatic degenerative disc disease with objective evidence of lumbar DDD, based on identification of any of the following characteristics by MRI scan:

◦ instability as defined by ≥ 3 mm translation or ≥ 5° angulation.

◦ osteophyte formation of facet joints or vertebral endplates.

◦ decreased disc height of > 2 mm as compared to the adjacent level.

◦ scarring/thickening of the ligamentum flavum, annulus fibrosis, or facet joint capsule.

◦ herniated nucleus pulposus.

◦ facet joint degeneration/changes.

◦ vacuum phenomenon.

• Single level symptomatic disease at L4/L5 or L5/S1.

• Minimum of six months of unsuccessful conservative treatment, including but not limited to physical therapy and/or medication.

• Minimum Oswestry Disability Index score of 40/100.

• Subject is a surgical candidate for an anterior approach to the lumbar spine.

• Back pain at the operative level only, with or without leg pain.

• Back pain, as measured using a visual analog scale (VAS), greater than the higher of the two VAS leg pain scores.

• Minimum VAS back pain score of 40/100 mm.

• Subject willing and able to return for follow-up visits regularly and sign an Informed Consent and HIPAA Authorization.

### Exclusion Criteria

• Previous surgery at any lumbar level, except IDET (Intradiscal Electrothermal Annuloplasty), percutaneous nucleoplasty, microdiscectomy, hemilaminectomy, or laminotomy

• Chronic radiculopathy as defined by subject complaint of unremitting pain with a predominance of leg pain symptoms greater than back pain symptoms extending over a period of at least 1 year.

• Anatomic requirements incompatible with the available range of dimensions for the experimental or control devices, based on preoperative assessment using radiographic templates. Specifically, endplate dimensions smaller than 34.5 mm in medial-lateral and/or 27 mm in the anterior-posterior direction

• Subjects with evidence of significant, symptomatic disc degeneration at another lumbar level.

• Preoperative remaining disc height < 3 mm

• Myelopathy.

• Previous compression or burst fracture at the affected level.

• Sequestered herniated nucleus pulposus with migration.

• Mid-sagittal stenosis of < 8 mm (by MRI).

• Degenerative or lytic spondylolisthesis > 3 mm.

• Spondylolysis.

• Isthmic spondylolisthesis.

• Lumbar scoliosis (> 11° sagittal plane deformity).

• Spinal tumor.

• Active systemic infection or infection at the site of surgery.

• Facet ankylosis or severe facet degeneration.

• Continuing steroid use or prior use for more than 2 months.

• History of allergies to any of the device components including cobalt chromium alloy, titanium, ultra high molecular weight polyethylene, and calcium phosphate.

• Pregnancy or planning to become pregnant within the next 2 years.

• Morbid obesity (BMI > 35).

• Investigational drug or device use within 30 days.

• Osteoporosis or osteopenia, indicated by a lumbar spine DXA T-score less than or equal to -1.

• Metabolic bone disease.

• Leg pain with migrated sequestrum fragment.

• History of rheumatoid arthritis, lupus, or other autoimmune disorder.

• Ankylosing spondylitis.

• History of HIV/AIDS or hepatitis that precludes surgery.

• History of deep vein thrombosis, symptoms of arterial insufficiency, or thromboembolytic disease.

• Current or recent history of illicit drug or alcohol abuse, or dependence as defined as the continued use of alcohol despite the development of social, legal, or health problems.

• Life expectancy less than 5 years.

• Undergone chemotherapy within the past 5 years, or had any cancer other than non-melanoma skin cancer treated with curative intent within the past 5 years.

• Prior nephrectomy.

• Abdominal adhesions, endometriosis, inflammatory bowel disease, Crohn's disease, diverticulitis, ulcerative colitis or other abdominal pathology that would preclude the abdominal surgical approach.

• Insulin-dependent diabetes.

• Any degenerative muscular or neurological condition that would interfere with evaluation of outcomes, including but not limited to Parkinson's disease, ALS (amyotrophic lateral sclerosis), or multiple sclerosis.

• History of Pelvic Inflammatory Disease.

• Peritonitis.

• Subjects currently in active spinal litigation as a result of medical negligence.

• Subject is a prisoner.

• Psychiatric or cognitive impairment that, in the opinion of the investigator, would interfere with the subject's ability to comply with the study requirements, e.g., Alzheimer's disease.

### Treatment Procedures and Evaluations

#### Activ-L Disc

All subjects randomized to receive the Activ-L device will first undergo discectomy to remove the damaged disc, then (in the same procedure) will be implanted with the device. The Activ-L disc is inserted completely assembled (endplates and inlay together) and implanted. No other implanted instrumentation is required to secure the device position.

#### Control Discs (ProDisc^®^-L Total Disc Replacement or Charité^® ^Artificial Disc)

All subjects randomized to receive one of the control devices (ProDisc-L or Charité) will first undergo discectomy to remove the damaged disc, then (in the same procedure) will be implanted with the device. Both the ProDisc-L and Charité implantations are achieved in two steps. The endplates are first positioned and inserted, followed by a second distraction step to permit insertion of the convex UHMWPE core or inlay between the endplates. No other implanted instrumentation is required to secure the device position. Please refer to table 1 for time and event schedules.

**Table 1:** Time and Events Schedule

	Base-line	Intra-OP	Discharge	Follow-up Period from Surgery Date (± Days)					
				6 wks (± 14)	3 mon (± 14)	6 mon (± 30)	12 mon (± 60)	24 mon (± 60)	3-5 yrs (± 60)
Inclusion/Exclusion Determination	**X**								
Osteoporosis/ osteopenia screen	**X**								
Medical History/Physical Exam	**X**								
Work Status	**X**			**X**	**X**	**X**	**X**	**X**	**X**
Pain Medications	**X**		**X**				**X**	**X**	
Antibiotics	**X**	X**	**X**						
Visual Analog Scale (VAS) Pain Assessment	**X**			**X**	**X**	**X**	**X**	**X**	**X**
Neurological Assessment	**X**		**X**	**X**	**X**	**X**	**X**	**X**	**X**
DVT Prophylaxis			**X**						
QOL SF-36	**X**			**X**	**X**	**X**	**X**	**X**	**X**
Oswestry Disability Index (ODI)	**X**			**X**	**X**	**X**	**X**	**X**	**X**
Hospital Stay			**X**						
Range of Motion	**X**					**X**	**X**	**X**	**X**
Subject Satisfaction							**X**	**X**	**X**
Adverse Events		**X**	**X**	**X**	**X**	**X**	**X**	**X**	**X**
**RADIOGRAPHIC EVENTS**									
MRI scan	**X**								
DXA Scan	**X (IR)**								
X-rays, A/P -- Standing Neutral	**X**	**X** **(Implant Position)**	**X** **(Implant Position)**	**X**	**X**	**X**	**X**	**X**	**X**
X-rays, A/P- Right/Left Bending	**X**			**X**	**X**	**X**	**X**	**X**	**X**
X-rays, Lateral - Flexion-Extension	**X**			**X**	**X**	**X**	**X**	**X**	**X**
X-Rays, Lateral Standing Neutral	**X**	**X** **(Implant Position)**	**X** **(Implant Position)**	**X**	**X**	**X**	**X**	**X**	**X**

IR: If required

** Prophylactic antibiotics

### Surgical Technique

A standard anterior retroperitoneal approach to the lower lumbar spine is utilized. A vascular access surgeon will be used in all cases. The implant comes in two versions: a keel and a spiked variant. The keel version is used in cases of extreme concavity of the endplate. In cases where the endplates are sclerotic, the spike version is indicated.

After the appropriate level is identified under image intensification, the midline of the vertebral body is determined. The spinous process and pedicles serve as rotational aids in determining the midline. Using a curette, the endplates are cleared of disc residue. A distractor is then used to mobilize the disc compartment. Under AP and lateral x-ray views, the trial plates are then inserted. Care is used to avoid placing the plates too deeply. The disc space is distracted to a point where the implant will be held firmly in place, the height measurement is then read off the distractor. The implant is then assembled on the back table and attached to the inserter. During implantation it is imperative that the implant does not deviate from a central position. The inserter is disconnected from the implant and the position of the implant is checked using image intensification.

Both treatment and control devices will be implanted anteriorly to ensure comparability between treatment groups. Blood loss is minimal, averaging approximately 50 cc.

### Intraoperative Data Collection

AP and lateral X-rays will be taken intra-operatively and post-operatively to verify prosthesis placement. Surgical data collected will include the date and duration of the surgery, level implanted, blood loss and prosthesis description. Surgical duration will be measured from the time of incision to the time of skin closure, skin-to-skin. All adverse events will be reported. Information will be collected during the surgical procedure and/or from the surgical record and will be documented on the Intra-operative Evaluation Form. The following information will be collected:

• Procedure date, time

• Prophylactic antibiotics

• Implanted device model number and lot number

• Surgery information including duration, approach, and use of an access surgeon

• Vertebral level treated

• AP and lateral X-rays to verify device placement

• Estimated blood loss

• Blood transfusion information (as needed)

• Adverse Events

• Any implant sizing difficulties

### Primary Endpoints

Individual subject success will be defined as:

• Improvement of at least 15 points in the Oswestry Disability Index score at 24 months compared to baseline.

• Maintenance or improvement in neurological status at 24 months compared to baseline as measured by motor and sensory evaluations. A decrease of one grade in either evaluation will be considered a failure.

• Maintenance or improvement in motion at the index level, defined as:

24 month ROM - preoperative ROM ≥ 0 (with ± 2° measurement error applied) in a patient who does not meet the definition of fusion.

Fusion as defined in the radiographic protocol will be considered a failure.

• No device failures requiring revision, re-operation, removal, or supplemental fixation.

• Absence of serious device-related adverse events (AEs). For purposes of this study, serious device-related adverse events are defined as serious device-related adverse events as adjudicated by the Clinical Events Committee.

For purposes of evaluating the primary endpoint, the Clinical Events Committee will determine if an adverse event is device-related. However, the investigator's assessment will also be reported.

For purposes of the above endpoint definition, the terms "revision," "re-operation," "removal," and "supplemental fixation" are defined as follows:

• A revision is a procedure that adjusts or in any way modifies or removes part of the original implant configuration, with or without replacement of a component. A revision may also include adjusting the position of the original configuration.

• A removal is a procedure where the entire original system configuration is removed with or without replacement.

• A re-operation is any surgical procedure at the involved level(s) that does not include removal, modification, or addition of any components to the system.

• A supplemental fixation is a spinal procedure in which additional instrumentation not under study in the protocol is implanted (e.g., supplemental placement of a rod/screw system or a plate/screw system). In addition, any subsequent procedures related to the index level should be reported. This would include posterior fusion, leaving or removing a motion retaining device in place, decompression, facet rhizotomy, etc at the same, adjacent or distant levels.

### Powered Secondary Endpoints

• Back Pain, measured at rest using a visual analog scale (VAS); improvement of 20 mm or more on a 100 mm VAS scale at 24 months compared to baseline will be considered clinically significant.

• Leg Pain, measured at rest using a visual analog scale (VAS). The success rate will be formally tested for superiority in the Activ-L group. A patient will be considered a responder if, at the 24 month visit they achieve at least a 20 mm VAS improvement in the leg with the maximum pain at baseline, with no worsening in the other leg.

### Unpowered Secondary Endpoints

• The mean Oswestry Disability Index score, as well as the mean improvement, will be compared between groups across all study visits. The incidence of both 15% and 15 point improvements will also be compared.

• Quality of Life, measured using the Short Form-36 Questionnaire (SF-36); improvement of 15% in the overall score at 24 months compared to baseline will be considered clinically significant.

• Disc height (incidence of 3 mm change), as measured by standard lateral radiograph. Comparison of disc height to the 6-week height will also be performed to account for the expected degree of immediate post-operative "settling" of the prosthesis.

• Incidence of subsidence of the device (> 3 mm) at the 24-month visit.

• Neurological status at 24 months compared to baseline.

• The mean operative time.

• Safety: The individual incidence rates of all adverse events through 24 months follow-up.

• Each subject will remain in the study for 5 years post treatment. It is expected to take 6 years to collect all required data for this study.

### Power Analysis

Power analysis was performed using the PASS 2005 software. (NCSS Kaysville, Utah)The PASS 2005 output solves for what the true success rate must be in the Activ-L group in order to have 80% power to detect superiority relative to a number of possible success rates in the control group (plausible range 50-80%). All calculations assume a power of 80% and a two-sided significance level of 0.05. Note that for each case, the required margin of improvement is not particularly onerous. For example, if the true success rate in the control group is 50%, the true success rate in the treatment group need only be 68%. If the control rate is 80%, the Activ-L rate need only be 92%.

Therefore, with the sample size required to demonstrate the primary composite endpoint, this study is adequately powered to detect a reasonable, yet clinically meaningful difference in rates if Activ-L truly produces greater improvement in pain over time. Please refer to table 2.

**Table 2:** Power Analysis results using PASS 2005 Software (NCSS Kayesville, Utah) PASS 2005 Power Analysis Results.

	Sample Size Group 1	Sample Size Group 2	Group 1 or Treatment	Group 2 or Control	Diff if H0	Diff if H1		
Power	N1	N2	P1.1	P2	D0	D1	Target Alpha	Beta
0.8000	178	89	0.6771	0.5000	0.0000	0.1771	0.0500	0.2000
0.8000	178	89	0.7227	0.5500	0.0000	0.1727	0.0500	0.2000
0.8000	178	89	0.7665	0.6000	0.0000	0.1665	0.0500	0.2000
0.8000	178	89	0.8086	0.6500	0.0000	0.1586	0.0500	0.2000
0.8000	178	89	0.8487	0.7000	0.0000	0.1487	0.0500	0.2000
0.8000	178	89	0.8865	0.7500	0.0000	0.1365	0.0500	0.2000
0.8000	178	89	0.9218	0.8000	0.0000	0.1218	0.0500	0.2000

Two Independent Proportions (Null Case) Power Analysis Numeric Results of Tests Based on the Difference: P1 - P2 H0: P1-P2 = 0. H1: P1-P2 = D1 <> 0. Test Statistic: Z test with pooled variance

### Follow Up Evaluations

Subjects will be followed at 6-weeks (± 14 days), 3-months (± 14 days), 6-months (± 30 days), 12-months (± 60 days) and 24-months (± 60 days). After completion of the 24-month follow-up, subjects will continue to be evaluated annually for 3 additional years (± 60 days) to gather additional long-term information. Information will be collected on the Follow-up Visit Form and the Individual forms for VAS, ODI and SF-36. The following information will be recorded as shown below.

Data collected at 6 weeks and 3 months will include:

• AP/Lateral, flexion/extension, and side bending standing x-rays

• Work status

• VAS Pain assessment (back, right and left leg)

• Oswestry Disability Index score

• Quality of life assessment (SF-36)

• Neurological assessment

• Adverse events

Data collected at 6 months will include:

• AP/Lateral, flexion/extension, and side bending standing x-rays

• Work status

• VAS Pain assessment (back, right and left leg)

• Oswestry Disability Index score

• Quality of life assessment (SF-36)

• Neurological assessment

• Range of Motion

• Adverse events

Data collected at 12 and 24 months will include:

• AP and Lateral, flexion/extension and lateral bending X-rays

• Work status

• VAS Pain assessment (back, right and left leg)

• Oswestry Disability Index score

• Quality of life assessment (SF-36)

• Neurological assessment

• Pain medication status

• Range of Motion

• Subject satisfaction

• Adverse events

The same follow-up information will be collected at subsequent annual visits through year 5 with the exception of the MRI scan and pain medication usage.

To collect data concerning the integrity of the blind, subjects will be asked at each follow-up visit if, since the prior visit, they have learned which device they received.

### Justification for Pooling Data across Centers

The proposed investigation is a multi-center trial in the US, where all centers will be trained to use the same study protocol. The appropriateness of pooling data from multiple centers is necessarily enhanced by training all participating centers to follow the same protocol, with clearly defined inclusion and exclusion criteria for study participation. However, if an interaction is present between treatment and center effects, the results will not be strictly poolable. Thus, in order to protect against improperly pooling data from all study centers, a formal analysis will be conducted to assess the appropriateness of pooling the data [8].

For the primary endpoint, a logistic regression will be constructed that models the success rate on three factors: treatment group, center, and the treatment group by center interaction. If the interaction term is significant at a two-sided α = 0.05 level of significance, then the offending center(s) will be identified and all analyses will be presented in a stratified fashion, since pooling the results would make interpretation of the trial difficult.

If, on the other hand, the interaction term is insignificant but the center term is significant, then fixed effects for center will be added to the final models for, at a minimum, the primary endpoint. Controlling for confounding center effects in this fashion is a well-accepted technique.

## Discussion

Present treatment for disc degeneration ranges from conservative modalities, such as rest, heat, electrotherapy, physical therapy, and analgesics to surgery. Currently, there are two main surgical techniques for treatment of disc degeneration: (1) nucleotomy or discectomy, i.e., excision of part or all of the degenerated disc, which is typically performed for treatment of radicular syndrome in the case of disc herniation; and (2) spinal fusion, i.e. grafting bone between the vertebrae adjacent to the degenerated disc to eliminate articulation at the damaged reduction of the intervertebral space. Rigid internal fixation may also be used to promote fusion.

The Activ-L Disc is designed to provide a new therapeutic option for treatment of degenerative disc disease as an alternative to spinal fusion to preserve function in the lumbar vertebral region. By allowing motion at the diseased segment the replacement disc would limit the advance of adjacent level disease as well as preclude the need for fusion.

The Activ-L, ProDisc-L and Charité devices are designed to provide a treatment modality as an alternative to fusion [9]. These devices are modular systems and are intended to be used as intervertebral dynamic disc spacers in the lumbar vertebral region at the L3/L4 (ProDisc-L only), L4/L5 and L5/S1 levels.

Our study will further allow the generation of results comparing fusions with the two types of disc replacements in treating degeneration in the lumbar spine. The results will have a relevant impact on providing additional safety and efficacy data in regards to a promising alternative to lumbar spine fusions.

## Competing interests

Dr James Yue is a consultant for Aesculap. Aesculap is the manufacturer of Activ-L Lumbar disc replacement and provided funding for this clinical trial.

## Authors' contributions

JY participated in the design and coordination of the study. FM drafted the manuscript. All authors read and approved the final manuscript.

## Pre-publication history

The pre-publication history for this paper can be accessed here:

http://www.biomedcentral.com/1471-2482/10/14/prepub
